# Exercise: a non-drug strategy of NK cell activation

**DOI:** 10.1590/1414-431X2024e14144

**Published:** 2024-11-25

**Authors:** Huixin Pan, Rui Meng, Zixuan Jia, Jing Zhang, Wen Ma, Youhan Liu, Qinglu Wang, Qiaoqiao Li

**Affiliations:** 1 College of Sport and Health, Shandong Sport University, Jinan, China; 2 School of Data Science, The Chinese University of Hong Kong, Shenzhen, China; 3 Key Laboratory of Biomedical Engineering & Technology of Shandong High School, Qilu Medical University, Zibo, China

**Keywords:** NK cell, Exercise, Immune system, Immune cell, Signaling pathway

## Abstract

Natural killer (NK) cells are a critical component of the innate immune system and one of the immune cells most sensitive to exercise. So far, it is widely believed that moderate exercise can significantly enhance the proliferation and activity of NK cells, strengthening immune function. However, the impact of exercise on NK cells is a dynamic and complex process. In addition to the type of exercise, the frequency, intensity, and duration of exercise are also key factors. This article not only briefly summarizes the activation mechanisms of NK cells but also delves into the potential importance of exercise as a non-pharmacological strategy in modulating NK cell activity and enhancing the immune system. Emerging studies have indicated that the timing and regularity of exercise bouts might also influence NK cell responses. Moreover, the interaction between exercise and other components of the immune system, such as cytokines and chemokines, could further modulate the functionality of NK cells. The above research is of crucial significance for achieving a deeper understanding of the intricate connection between exercise and NK cell function, as well as the development of effective health promotion strategies. In addition, further research is needed to investigate the effects of long-term exercise on NK cell function and the interaction between exercise and NK cell-mediated immune responses. Translating these research findings into precisely tailored exercise programs for specific populations, taking into account factors like age, health status, and genetic predisposition, could potentially offer unprecedented prospects for further advancements in this burgeoning field of study.

## Introduction

Exercise has long been recognized as one of the most important factors in maintaining good health. Recent research shows that exercise benefits the cardiovascular, musculoskeletal, and immune systems. The impact on the immune system largely depends on the type, intensity, and duration of exercise (1). Various studies have demonstrated that regular physical activity can enhance the body's immune function, reduce the risk of chronic diseases, and improve overall health and quality of life. In addition to this, exercise acts as a modulator of the immune system, allowing it to produce a more regulated immune response and improve inflammation (2). Not only does it have a positive effect on the immune organs, but it also enhances the vitality of immune cells and immune factors and reduces inflammatory markers (cystatinase-3, lactate dehydrogenase, and nitric oxide) and inflammatory cytokines (interleukin (IL)-1b, IL-6, IL-8, and tumor necrosis factor alpha (TNF-α)) (3).

Among other things, exercise generates a systemic response in the immune system, and immune cell populations have been observed to respond to exercise stimuli, especially natural killer (NK) cells, which are among the immune cells most responsive to exercise and whose numbers begin to increase within a few minutes after exercise begins (2). This rapid response suggests that NK cells play a crucial role in the body's immediate defense against pathogens and abnormal cells. NK cells are important immune cells in the body and constitute the first line of defense when the body is infected by viruses and tumor cells (4). They can recognize and kill infected or cancerous cells without prior activation, providing a rapid and effective immune response. As a distinct population of cells of the innate immune system, NK cells are characterized by rapid activation via germline-encoded receptors that differentiate between healthy and diseased cells, resulting in spontaneous killing of tumor cells and infectious pathogens (5). NK cells respond strongly to exercise, especially acute exercise, mainly by changes in their number and function. The aim of this review is to explore the molecular mechanisms of NK cell activation by different exercise types.

## Basic characteristics of NK cells

### Classification and biological functions of NK cells

NK cells are a subpopulation of lymphocytes that play an important role in the immune system, and were first observed in 1975 by the American immunologist Ervin Podack. NK cells have a variety of biological functions, mainly including direct killing of tumor cells and virus-infected cells, production of cytokines to regulate the immune response, and participation in the formation of immune memory, so NK cells play a key role in the immune system (5). The CD56 marker divides NK cells into two major subsets: CD56bright and CD56dim (6). The CD56bright subset mainly exerts the function of immune regulation by secreting cytokines; the CD56dim subset usually has stronger cytotoxicity and can directly kill tumor cells or infected cells, while the cytotoxicity of the CD56bright subset is relatively weaker.

NK cells have direct cytotoxic effects against tumor cells and virus-infected cells. They interact with target cells through their surface activation receptors, releasing perforin and granzymes to disrupt the membrane structure of target cells and induce apoptosis (5). In addition to their killing role, NK cells also play an important role in immunomodulation, mainly through the production of cytokines such as interferon-γ (IFN-γ) (7). IFN-γ activates macrophages, enhances their function, and promotes T-cell activation, which affects immune cell differentiation and function. NK cells also produce other cytokines such as TNF-α, which work together to maintain immune homeostasis. An in-depth understanding of these functions and their molecular mechanisms is important for fully exploiting the role of NK cells in immune defense.

### The role of NK cells in immune surveillance

NK cells play a key role in immune surveillance, especially in defending against tumors and viral infections. They intervene early, identifying and eliminating cells that have transformed into tumor cells or become infected, releasing perforin and cytokines to destroy abnormal cells, thereby halting the progression of the disease (4). In tumor immune surveillance, NK cells disrupt the membrane structure of cancer cells, induce apoptosis, and inhibit their growth by releasing perforin and cytokines such as granzymes and IFN-γ (8). This direct anti-tumor effect makes NK cells an important target for immunotherapy. In defending against viral infections, NK cells first recognize viral pathogen components or stress signals through surface activating receptors (such as NKG2D and DNAM-1 receptors), while inhibitory receptors recognize “self” to avoid mistakenly killing healthy cells. During a viral infection, NK cells are directly activated by inflammatory cytokines. Upon encountering a potential target, they integrate signals to determine their actions. Once activated, NK cells can release granzymes and perforin to lyse target cells, produce cytokines like IFN-γ to alert surrounding tissues of the infection, and potentially proliferate. NK cells exhibit great diversity to adapt to viral changes, with NK cell receptors of different species evolving in various ways; in some animal hosts, their activation thresholds are tightly regulated (9).

In addition, NK cells interact with other immune cells to regulate the balance of the immune response (10). By producing cytokines, NK cells influence the activity of immune cells, such as T lymphocytes and macrophages, to maintain the normal function of the immune system. Overall, in-depth studies of the molecular mechanisms of NK cells in immune surveillance are important for understanding immune regulation and developing relevant therapeutic regimens.

## Mechanisms of NK cell activation

### NK cell activation and inhibitory receptors

Receptors on the surface of NK cells play a crucial role in the regulation of their function. These receptors can be categorized into two main groups - activating receptors and inhibitory receptors (11).

Activated receptors on the upper surface of NK cells include the natural killer group 2, member D (NKG2D) receptor and the DNAX accessory molecule-1 (DNAM-1) receptor (12). NKG2D receptor is an important activating receptor that interacts with ligands such as MICA/MICB and ULBP to trigger NK cell activation. Exercise increases the expression of the NKG2D receptor, which enhances the activation potential of NK cells; DNAM-1 receptor binds to its ligands CD155 and CD112, contributing to the enhanced killing of target cells by NK cells (13). Exercise helps to enhance the expression of DNAM-1 receptor, which in turn promotes the activation of NK cells.

In addition to activating receptors and inhibitory receptors, NK cells also express other important receptors, such as those of the adrenergic receptor (AR) family, which are closely related to the regulation of NK cell function. For example, norepinephrine (NE) can inhibit the cytotoxicity of NK92-MI cells through the β2-adrenergic receptor (β2-AR)/cyclic AMP (cAMP)/protein kinase A (PKA)/phosphorylated cAMP response element-binding protein (p-CREB) signaling pathway, reducing the expression of perforin, granzyme B, and IFN-γ (14). Moreover, receptors like NKP30, NKP44, NKP46, and TNFR1 play roles in recognizing cells infected by certain viruses.

Inhibitory receptors on NK cells include KIR receptors and NKG2A receptors. The KIR (killer cell immunoglobulin-like receptor) family is a group of inhibitory receptors that interact with HLA-like molecules for recognizing their own cells (15). By exercising, the inhibitory effect of the KIR receptor can be attenuated, making it easier for NK cells to be activated. The NKG2A receptor interacts with its ligand, HLA-E, and acts to inhibit NK cell activity (16). Exercise reduces NKG2A receptor expression, which enhances NK cell activation.

### Activation of signaling pathways

#### Structure and function of activation receptors

Receptors for NK cell activation are diverse, and two of the major ones include the NKG2D receptor and the DNAM-1 receptor (17). NKG2D receptor is a C-type lectin-like receptor that belongs to one of the major activating receptors for NK cells. The structure of this receptor consists of an extracellular portion and an intracellular portion. The extracellular portion contains two C-type lectin structural domains for interaction with its ligands. The ligand for the NKG2D receptor include MICA, MICB, and ULBP, which are overexpressed on infected and tumor cells (18). When the extracellular portion of the NKG2D receptor binds to the ligand, its intracellular portion transmits an activation signal via the DAP10 adapter protein (19). This activation signal triggers the killing of target cells by NK cells. The DNAM-1 receptor is an immunoglobulin superfamily member with two Ig-like structural domains (20). DNAM-1 binds to ligands on disease and tumor cells, prompting NK cells to respond to these abnormal cells.

Inhibitory receptors of NK cells play a role in balancing and regulating their activity, mainly including KIR receptors and NKG2A receptors (21). The KIR family is a diverse group of inhibitory receptors that recognize molecules of the major histocompatibility complex (MHC). By interacting with MHC molecules, KIR receptors recognize their own cells and prevent NK cells from attacking their own tissues. However, certain viral infections and tumors can lead to loss of MHC molecules, which attenuates the inhibitory effect of the KIR receptor and allows NK cells to generate a stronger immune response to infected cells. The NKG2A receptor is another inhibitory receptor that interacts with the molecule HLA-E. HLA-E is part of the MHC class I molecules that are expressed on the surface of many cells (22). When NKG2A receptors bind to HLA-E, NK cell activity is inhibited. However, certain pathogenic infections or tumors can lead to reduced expression of HLA-E, which attenuates the inhibitory effect of the NKG2A receptor and thus enhances NK cell activation (23).

#### Activation of signaling pathways

In exploring the mechanisms of NK cell activation, we need to delve into the activation signaling pathways that are closely related to these processes. The PI3K/AKT signaling pathway plays a key role in the activation of NK cells. Activation of this signaling pathway is usually triggered by the binding of activating receptors on NK cells, such as NKG2D and DNAM-1, to their corresponding ligands (24). When the activation receptor of an NK cell is activated by its ligand, PI3K is activated and begins to catalyze the conversion of phosphatidylinositol bisphosphate (PIP2) to phosphatidylinositol triphosphate (PIP3) on the cell membrane (25). This step involves the class I isoforms of PI3K, specifically p110γ and p110δ. Generation of PIP3 leads to migration of the AKT kinase to the cell membrane and activation by phosphorylation (26). Activated AKT further phosphorylates multiple substrates, including BAD (Bcl-2-associated death promoter) and GSK-3β (glycogen synthase kinase-3β), to promote cell survival and proliferation (27). A downstream effect of the PI3K/AKT signaling pathway is the activation of mTORC1 (mammalian target of rapamycin complex 1). mTORC1 contributes to protein synthesis and cell growth by phosphorylating and regulating its substrates, such as S6K1 (ribosomal protein S6 kinase 1) and 4E-BP1 (eukaryotic translation initiation factor 4E-binding protein 1) (28).

The ERK/MAPK signaling pathway also plays an important role in NK cell activation. This signaling pathway is often mediated by multiple activating receptors and plays a key role in eliciting cellular stress responses and cell proliferation (29). Activation receptors on NK cells (e.g., NKG2D and DNAM-1) trigger downstream signaling upon binding to their ligands. This usually involves activation of the Ras protein family. Activated Ras transduces signals by activating Raf kinase (Raf-1) (30). Next, Raf-1 activates MEK (MAPK/ERK kinase) kinase, which ultimately leads to phosphorylation and activation of ERK (extracellular signal-regulated kinase). The activated ERK kinase can phosphorylate multiple substrates, including transcription factors ELK-1, c-Myc, c-Fos, etc., thereby regulating gene expression and cell proliferation (31).

The calcium signaling pathway is crucial for NK cell activation, especially in terms of intercellular interactions and cytotoxicity. Upon receptor activation, intracellular calcium ions are released from cytoplasmic calcium stores (such as the endoplasmic reticulum and mitochondria), leading to an increase in intracellular calcium concentration (32). This will trigger a series of signaling cascades, including the activation of calcium/calmodulin-dependent protein kinases (CaMKs). The activation of the calcium signaling pathway prompts NK cells to execute various effects, including intercellular adhesion, target cell release, and cell killing (32).

Additionally, the PKC and PKA signaling pathways also play significant roles in NK cell activation. The PKC signaling pathway can enhance the proliferation of splenic lymphocytes and NK cell activity, contributing to immune regulation (33). The PKA signaling pathway is involved in the inhibition of NK cell cytotoxicity by norepinephrine (14).

**Figure 1:**
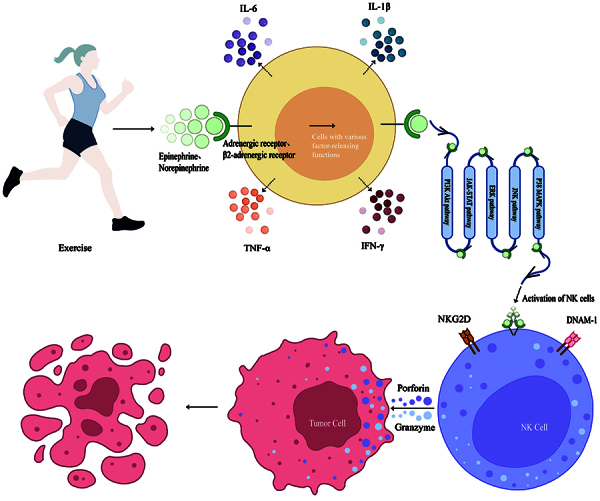
Exercise can promote the release of various cytokines and inflammatory factors such as interleukin (IL)-6, tumor necrosis factor (TNF)-α, interferon (IFN)-γ, and IL-1β. In addition, exercise can also promote the release of neurotransmitters such as adrenaline and noradrenaline. Adrenaline and noradrenaline bind to adrenergic receptors and β2-adrenergic receptors. After receptor activation, the signal is transmitted through intracellular signaling pathways such as PI3K-Akt, ERK, JNK, p38 MAPK, and JAK-STAT into the interior of natural killer (NK) cells. The activating receptors on the surface of NK cells include NKG2D and DNAM-1, among others. When these receptors bind to their ligands, NK cells are activated, initiating signal transduction pathways. NK cells contain abundant perforin and granzymes, which can eliminate tumor cells.

Understanding these signaling pathways is key to deciphering how NK cells activate and their role in immune responses (Figure 1). The effect of exercise on these pathways may influence NK cell function, a potential research area for developing immune enhancement strategies (34).

## Effects of exercise on NK cells

### Effects of exercise on the immune microenvironment of NK cells

Research has shown that exercise significantly enhances the activity of NK cells, with exercise-induced enhancement of NK cell activity primarily manifested in increased cytotoxicity (35). This means that after exercise, NK cells can more effectively recognize and eliminate cells that pose potential harm. This effect involves the interaction of various factors, including hormonal regulation, cytokine production, and activation of cell surface receptors (36).

Exercise induces changes in hormone levels in the body, especially an increase in catecholamines, which are adrenaline-related chemicals released during exercise, activating NK cells. IFN-γ plays a crucial role in this process: it is a typical product of NK cells and is essential for antiviral and antitumor immunity (7). Exercise enhances NK cell activity by activating NK cell surface receptors such as NKG2D, NKp30, NKp44, etc. These receptors may interact more readily with target cells during exercise, thereby enhancing cytotoxic effects (37).

Exercise can alter the distribution of NK cell subsets, particularly by increasing the proportion of the CD56 subset (38). Exercise can lead to an increase in the CD56dim subset (39), but its specific mechanism is still unclear. The speculated possible mechanisms are as follows: exercise promotes the mobilization of lymphocytes from secondary lymphoid tissues to peripheral tissues, which may lead to an increase in the CD56dim subset; exercise has an impact on the neuroendocrine system, causing an increase in the secretion of hormones such as epinephrine, which may play a role in the mobilization and distribution of CD56dim cells; due to the stronger cytotoxicity of CD56dim cells, exercise may regulate the function of immune cells, enabling them to play a more important role in the immune response, thereby leading to an increase in their number.

Research suggests that besides altering the immune microenvironment of NK cells, different types, intensities, and durations of exercise have significant effects on the quantity, activity, and adaptive changes of NK cells (40-44). Various forms of exercise, such as aerobic exercise and swimming, have diverse impacts on NK cell function. Additionally, the intensity and duration of exercise are key factors determining the NK cell response.

### Effects of type and intensity of exercise on NK cells

#### Effects of aerobic exercise on NK cells

Aerobic exercise, as a widely studied type of physical activity, holds profound significance for the immune impact on NK cells. This form of exercise involves prolonged periods of low to moderate-intensity physical activities, such as jogging, swimming, and cycling. Aerobic exercise has profound effects on the immune system.

Firstly, aerobic exercise helps to increase the activity of NK cells (45). This effect is partly attributed to the release of hormones in the body, with adrenaline and noradrenaline playing crucial roles in the activation process of NK cells. Secondly, aerobic exercise plays a significant role in the migration and distribution of immune cells. During exercise, NK cells migrate from internal body regions, including the circulatory system, lymphatic system, and interstitial spaces, to specific locations such as exercising muscles and lymphoid tissues, primarily through chemotaxis mechanisms. Chemotactic factors, such as cytokines and chemical mediators, are produced during exercise and disseminate in the bloodstream (46). They originate from various cells, including immune cells, muscle cells, and endothelial cells. These factors are generated in response to the physiological stress and metabolic changes induced by physical activity. They function by binding to G protein-coupled receptors (GPCRs), attracting immune cells to migrate towards specific locations (47). Immune cells perceive chemotactic factors through their surface receptors, triggering signaling pathways including phosphorylation cascades, activation of intracellular signaling molecules, and regulation of gene expression. These signaling pathways enable immune cells to sense immune threats and mount migratory responses (48).

Furthermore, aerobic exercise also has a positive impact on the overall efficiency of the immune system (49). Aerobic exercise promotes interactions between immune cells, particularly the synergistic interactions between NK cells and other immune cells such as T cells and macrophages (50). This synergy is achieved through cell adhesion and intercellular signal transduction.

Cell adhesion involves physical connections between immune cells or between immune cells and the vessel wall, which are critical for their migration, localization, and immune responses (51). Aerobic exercise enhances the adhesion and recruitment ability of immune cells by increasing the expression of adhesion molecules such as integrins and selectins (52). This allows immune cells to effectively reach and act upon sites of infection or injury, facilitating pathogen recognition and clearance.

Intercellular signal transduction involves immune cells communicating via chemical signals such as cytokines. These signals bind to receptors, triggering intracellular responses that influence cell activation, proliferation, and differentiation (53). Exercise has been shown to alleviate inflammatory states. During exercise, increased blood flow velocities induce shear stress, prompting the production of anti-inflammatory molecules like IL-10, TNF-α inhibitors, and transforming growth factor-beta (TGF-β) within endothelial cells lining blood vessels. This response helps promote anti-inflammatory effects (54,55).

Aerobic exercise regulates the production of these signaling molecules, elevating levels of anti-inflammatory cytokines, thus preventing tissue damage caused by excessive inflammation, which refers to an uncontrolled or disproportionate immune response characterized by an overproduction of inflammatory mediators. This can lead to tissue damage, organ dysfunction, and other adverse effects on the body (56).

In summary, aerobic exercise affects NK cell immune responses through multiple pathways, including directly enhancing activity, promoting immune cell migration and distribution, and enhancing the overall efficiency of the immune system. These immune effects provide a solid scientific basis for the application of aerobic exercise as an immunomodulatory strategy, while also highlighting the complex and important relationship between exercise and the immune system (57).

#### Effects of acute exercise on NK cells

Acute exercise, such as short-distance running and high-intensity interval training (HIIT), is a form of short-term, high-intensity physical activity that has positive effects on cardiovascular health and the immune system (58). Following acute exercise, the heart pumps blood more efficiently, delivering oxygen and nutrients throughout the body during and after exercise, and effectively distributing immune cells from the bone marrow and lymphoid organs, thereby enhancing NK cell activity and efficacy (59).

High-intensity acute exercise can trigger immune responses as well as immune suppression. High-intensity acute exercise leads to a sharp increase in adrenaline and noradrenaline levels in the body. These two hormones are part of the stress response, released rapidly at the onset of exercise to meet the body's demands. Adrenaline and noradrenaline can interact with receptors on the surface of NK cells, thereby triggering a series of signal transduction pathways (60). These pathways include the calcium signaling pathway, the protein kinase C (PKC) pathway, and the protein kinase A (PKA) pathway. Among the receptors expressed on the surface of NK cells, the most important is the adrenergic receptor (AR) family. This family includes two main classes: α-adrenergic receptors (α-AR) and β-adrenergic receptors (β-AR), corresponding to α-AR and β-AR subtypes, respectively.

Firstly, activation of α-AR and β-AR can lead to an increase in intracellular calcium ion (Ca^2+^) concentration, thereby influencing NK cell function through the activation of the calcium signaling pathway (61,62). These pathways include calcium/calmodulin-dependent protein kinase (CaMK) and phospholipase C (PLC). The increase in Ca^2+^ concentration triggers the activation of CaMK, which in turn initiates phosphorylation of a series of downstream signaling molecules, ultimately affecting the activity of NK cells. Meanwhile, activation of PLC leads to the hydrolysis of phosphatidylinositol (PI), generating inositol trisphosphate (IP_3_), which subsequently releases stored intracellular Ca^2+^. The activation of these signaling pathways promotes the activation and enhancement of NK cell function (63).

The action of adrenaline also involves the PKC and PKA pathways. PKC and PKA are important intracellular protein kinases that also play crucial roles in NK cells. Adrenaline can activate the PKC and PKA pathways by activating α-AR and β-AR (64). Activation of the PKC pathway leads to phosphorylation of a series of substrate proteins, including calmodulin-binding proteins and phospholipase A_2_ (PLA_2_), which further impact the activity of NK cells (65). Activation of the PKA pathway regulates the gene expression and function of NK cells through phosphorylation of intracellular proteins such as cAMP response element-binding protein (CREB). The activation of these signaling pathways ultimately leads to the activation of NK cells and enhances their cytotoxic function. This acute activation helps to increase NK cell cytotoxicity against potential pathogens or abnormal cells (66,67).

High-intensity acute exercise also promotes the production of more immune regulatory factors in the body, such as IL-6 and TNF-α (68). The release of IL-6 and TNF-α is a typical part of the inflammatory response, and they can affect NK cell activity through multiple pathways (69,70). Among them, IL-6 is believed to directly stimulate the activity of NK cells, enhancing their cytotoxicity, while TNF-α can activate NK cells in certain situations, especially in inflammatory environments (71). The release of these immune regulatory factors further supports the immune function of NK cells. Research indicates that compared to long-duration, high-intensity acute exercise, moderate-duration (<60 min) and intensity (<60% VO_2_max) acute exercise imposes less disruption and stress on the immune system (72).

The immune effects of acute exercise are two-sided. On the one hand, acute exercise can trigger immune responses, activate NK cells, and enhance the immune system's responsiveness. On the other hand, acute exercise may also lead to short-term immune suppression, with mechanisms including cortisol release induced by high-intensity exercise (73). Elevated levels of cortisol suppress the proliferation and function of NK cells, weakening their activity (74). Additionally, high-intensity acute exercise may lead to electrolyte imbalance in the body, such as loss of sodium and potassium. Electrolyte imbalance may weaken the activity of NK cells, reducing their efficacy in immune surveillance and clearing foreign substances, resulting in impaired immune responses (75). This short-term immune suppression and electrolyte imbalance represent potential negative effects of acute exercise on the immune system, necessitating appropriate recovery and supplementation post-exercise to maintain normal immune function (76).

Overall, the impact of high-intensity acute exercise on NK cells is a dynamic and complex process, involving various physiological and immunological mechanisms. This includes changes in hormone levels, release of immune regulatory factors, activation of signaling pathways, and alterations in immune cell function. While acute exercise may lead to immune suppression to some extent, it can also support the function of the immune system by activating NK cells and enhancing their cytotoxic function. When delving into the interaction between acute exercise and the immune system, it is necessary to consider these complex mechanisms comprehensively.

#### Effects of exercise intensity on NK cells

In addition to the type of exercise, the intensity of exercise is also a key factor influencing NK cells. Low-intensity exercise, such as casual walking or gentle jogging, imposes less stress on the body and can be sustained. Compared to moderate or high-intensity exercise, the impact of low-intensity exercise on NK cells is relatively limited (43).

Low-intensity exercise may fail to induce significant inflammation response within the body, and the relatively lower levels of cytokine release may not provide sufficient signaling stimuli to activate NK cells. As the enhancement of immune activity typically requires cytokine release, the amount of cytokines induced by low-intensity exercise is relatively small (77). Secondly, the activity of NK cells is regulated by multiple receptors, such as NKG2D receptor, NKp30, and NKp46, which play crucial roles in sensing target cells or pathogens. However, low-intensity exercise may not provide sufficient activation signals to these receptors, thus failing to fully activate NK cells. In the context of low-intensity exercise, the reduction in intercellular interactions results in fewer immune regulatory signals received by NK cells. In summary, the impact of low-intensity exercise on NK cells is mainly characterized by a relatively lower enhancement of immune activity. Compared to moderate or high-intensity exercise, low-intensity exercise induces lower levels of cytokine release and receptor signal activation, with a reduction in intercellular interactions (77). Nevertheless, low-intensity exercise still holds certain value in maintaining the fundamental functions of the immune system and immune health. Future research could delve deeper into the molecular mechanisms between low-intensity exercise and NK cells to comprehensively understand its impact on the immune system, providing more accurate guidance for exercise prescriptions and immune regulation.

Moderate-intensity exercise, typically including activities such as brisk walking, leisurely cycling, swimming, and others, can alleviate inflammation in the body (78). This inflammatory response is achieved through the production and release of various cytokines and immune modulatory substances, including TNF-α, IFN-γ, and others. These molecules are activated during moderate-intensity exercise, forming a complex immune regulatory network in which NK cells are an important component (79). During moderate-intensity exercise, the release of cytokines such as TNF-α can directly stimulate the activity of NK cells by activating receptors on the surface of NK cells, such as TNFR1. This process involves the activation of signaling pathways such as NF-κB and MAPK, ultimately resulting in the activation of NK cells and enhancement of cytotoxicity (80). Furthermore, the release of IFN-γ also plays a crucial role in the function of NK cells, as IFN-γ not only promotes the proliferation and activity of NK cells but also enhances their ability to recognize and kill target cells. Moderate-intensity exercise helps enhance NK cells' recognition and killing ability against tumor cells and viral infections, involving increased expression and affinity of NK cell surface receptors, thus strengthening the interaction between NK cells and target cells. The high cytotoxic activity of NK cells is crucial for the clearance of early-stage cancer cells and the control of pathogen infections (43).

High-intensity exercise typically includes prolonged, vigorous activities such as intense long-distance running, high-intensity interval training, and others, characterized by inducing drastic physiological changes and inflammation responses within a short period. However, high-intensity exercise may trigger complex dynamic changes in the immune system, including short-term activation of NK cells and long-term immune suppression (42,44). Firstly, high-intensity exercise may acutely activate NK cells in the short term, which is due to the inflammation response induced by exercise, including the release of cytokines such as TNF-α and IFN-γ. These factors can stimulate NK cell receptors, such as TNFR1, activating NK cells. Meanwhile, high-intensity exercise triggers the sympathetic nervous system, leading to the release of adrenaline and cortisol, further promoting NK cell activity (73). However, while high-intensity exercise can temporarily enhance the cytotoxicity of NK cells, the stress response it triggers may elevate cortisol levels, potentially leading to long-term immune suppression, including a decrease in NK cell function (81). This means that although high-intensity exercise can initially activate NK cells temporarily, it also comes with the risk of immune function decline. High-intensity exercise may have a lasting impact on the long-term immune activity and quantity of NK cells. Some studies have found that individuals who regularly engage in high-intensity exercise may experience chronic inflammation responses and persistent immune suppression, affecting NK cell function. Additionally, prolonged periods of high-intensity exercise may lead to a temporary decrease in NK cell count, especially during the recovery period, but this is typically transient and can recover after adequate rest (82).

### Effects of exercise duration and frequency on NK cells

#### Effects of exercise duration on NK cells

Different durations of exercise elicit different physiological and immune responses. Short-duration exercise has limited effects on NK cells because it does not cause significant physiological changes and immune stress. Following short-duration exercise, the immune activity of NK cells may temporarily increase, but due to low levels of cytokine release, this activation quickly returns to baseline levels thereafter (83).

Moderate-duration exercise, such as moderate-paced running or cycling, has a more prolonged effect on NK cells. This type of exercise can elevate body temperature, thereby enhancing the metabolic activity and cytokine production of NK cells (84). Meanwhile, the sustained inflammation response triggered by exercise, through cytokines produced by muscle tissue (such as TNF-α and IL-6), stimulates the activity of NK cells, enhancing their ability to surveil and eliminate potential threats. Furthermore, the inflammatory response promotes the proliferation and patrol of NK cells, strengthening immune function. The immune system may also adapt to the physiological changes induced by exercise, such as cytokine release and cellular metabolism adjustments, making NK cells more sensitive and efficient in responding to infections and diseases (85).

The impact of long-duration exercise on NK cells is more complex than short or moderate-duration exercise, involving interactions of multiple physiological and immune mechanisms, with profound effects on NK cell immune activity, including both active activation and potential suppression. Long-duration exercise may result in sustained physiological stress and inflammation response within the body. This type of exercise activates cardiovascular, respiratory systems, and metabolic pathways, requiring a significant amount of energy and oxygen, which triggers inflammation and the release of cytokines such as IL-6 and TNF-α. These factors stimulate NK cell immune activity in the short term, enhancing their ability to surveil and eliminate potential threats (72). However, long-duration exercise also activates anti-inflammatory mechanisms, such as cortisol release, which may suppress the immune system, including the activity of NK cells. Following long-duration exercise, NK cell function may decrease due to the activation of anti-inflammatory mechanisms during exercise, aimed at limiting excessive immune activation and inflammation. Additionally, long-duration exercise may lead to immune system fatigue, which is a state of immune suppression. Due to the prioritization of resource allocation over exercise performance during exercise, the operation of the immune system may be inhibited, increasing the risk of infection and immune related diseases in the body (86).

The duration of exercise has varying effects on NK cell immune activity: short-duration exercise has limited lasting effects on NK cell immune activity, moderate-duration exercise may enhance the baseline activity of NK cells, while long-duration exercise may lead to immune suppression. These effects involve various physiological and immune mechanisms such as body temperature, cytokines, and stress hormones. Future research should delve deeper into the molecular and cellular mechanisms underlying these effects to comprehensively understand the relationship between exercise duration and NK cells, providing guidance for exercise prescriptions and immune regulation (84).

#### Effects of exercise frequency on NK cells

The immune effects of low-frequency exercise are typically weaker than those of high-frequency and moderate-frequency exercise (87). Due to the lower volume and intensity of low-frequency exercise, it is insufficient to trigger significant physiological changes and immune stress, resulting in weaker activity, cytotoxicity, and cytokine production of NK cells. Additionally, the release of cytokines such as IL-6 and TNF-α induced by low-frequency exercise is relatively minimal, thus resulting in weaker activation effects on NK cells. After the cessation of exercise, the activity of NK cells may quickly return to baseline levels, indicating that their immune effects are not long-lasting (88).

Moderate-frequency exercise induces physiological changes such as increased body temperature, accelerated cardiovascular activity, and short-term inflammation responses, leading to the acute activation of NK cells to a highly active state (89). It also results in the release of cytokines such as IL-6 and TNF-α, stimulating NK cell immune activity, enhancing the response to threats to the body. The immune system may gradually adapt to these changes, making NK cells more sensitive and efficient in responding to infections and diseases. Unlike low-frequency exercise, the immune effects of moderate-frequency exercise are more enduring, with NK cell activity potentially remaining elevated after exercise, contributing to the maintenance of immune health (84).

High-frequency exercise may have adverse effects on NK cells. Frequent vigorous exercise leads to significant physiological changes, cytokine release, and excessive inflammation responses, burdening the immune system and potentially causing overactivation. This type of exercise may also result in immune system fatigue, suppressing immune function and NK cell activity, thereby reducing the efficiency of NK cells in surveilling and eliminating potential threats (90).

In summary, the duration and frequency of exercise significantly impact NK cell immune activity. Moderate-duration and frequency exercise may enhance the baseline activity of NK cells, while excessive or frequent exercise may lead to immune suppression. These effects involve various physiological and immune mechanisms such as body temperature, cytokines, and stress hormones.

## Conclusion

This review demonstrates how exercise activates NK cells through molecular mechanisms, revealing its association with immune function. Research indicates that exercise not only increases the number and activity of NK cells but also enhances the body's defense against pathogens and cancer by influencing cellular signaling pathways and cytokine expression. We found that different types and intensities of exercise have varying effects on NK cell activation, with aerobic exercise and intermittent high-intensity exercise being particularly effective, providing a basis for developing specific exercise regimens.

In addition, we also face several key research areas that remain unclear. Regarding the specific effects of the type and intensity of exercise on NK cell activation, future studies should focus on elucidating the detailed mechanisms underlying these variations. For example, investigating how different exercise modalities affect the expression and activation of specific receptors on NK cells, as well as the downstream signaling pathways involved. Additionally, exploring the optimal combination of exercise types and intensities to maximize NK cell activation and immune function would be valuable. The role of individual differences such as age, gender, and genetic background in the process of exercise-induced NK cell activation also warrants further investigation. Understanding how these factors modulate the response of NK cells to exercise could lead to personalized exercise recommendations based on an individual's characteristics.

Further research is needed on the long-term effects of exercise on NK cell function, especially its role in chronic health and disease prevention. Longitudinal studies tracking the changes in NK cell function over an extended period of time would provide valuable insights into the sustained benefits of exercise on the immune system. Additionally, investigating the interaction between exercise and NK cell-mediated immune responses in the context of specific diseases or conditions would help identify targeted exercise interventions for disease prevention and management.

Transforming these findings into exercise programs tailored for specific diseases or populations is also a challenge. Future research should focus on developing practical guidelines and protocols for implementing exercise interventions that can effectively modulate NK cell function and improve health outcomes. These studies are crucial for a deeper understanding of the connection between exercise and NK cell function and for developing effective health promotion strategies.
